# Self-assembled nanoparticle-enzyme aggregates enhance functional protein production in pure transcription-translation systems

**DOI:** 10.1371/journal.pone.0265274

**Published:** 2022-03-17

**Authors:** Meghna Thakur, Joyce C. Breger, Kimihiro Susumu, Eunkeu Oh, Joseph R. Spangler, Igor L. Medintz, Scott A. Walper, Gregory A. Ellis

**Affiliations:** 1 Center for Bio/Molecular Science and Engineering, Code 6900, U.S. Naval Research Laboratory, Washington, District of Columbia, United States of America; 2 College of Science, George Mason University, Fairfax, Virginia, United States of America; 3 Optical Sciences Division, Code 5600, U.S. Naval Research Laboratory, Washington, District of Columbia, United States of America; 4 Jacobs Corporation, Dallas, Texas, United States of America; University of Newcastle, AUSTRALIA

## Abstract

Cell-free protein synthesis systems (CFPS) utilize cellular transcription and translation (TX-TL) machinery to synthesize proteins *in vitro*. These systems are useful for multiple applications including production of difficult proteins, as high-throughput tools for genetic circuit screening, and as systems for biosensor development. Though rapidly evolving, CFPS suffer from some disadvantages such as limited reaction rates due to longer diffusion times, significant cost per assay when using commercially sourced materials, and reduced reagent stability over prolonged periods. To address some of these challenges, we conducted a series of proof-of-concept experiments to demonstrate enhancement of CFPS productivity *via* nanoparticle assembly driven nanoaggregation of its constituent proteins. We combined a commercially available CFPS that utilizes purified polyhistidine-tagged (His-tag) TX-TL machinery with CdSe/CdS/ZnS core/shell/shell quantum dots (QDs) known to readily coordinate His-tagged proteins in an oriented fashion. We show that nanoparticle scaffolding of the CFPS cross-links the QDs into nanoaggregate structures while enhancing the production of functional recombinant super-folder green fluorescent protein and phosphotriesterase, an organophosphate hydrolase; the latter by up to 12-fold. This enhancement, which occurs by an undetermined mechanism, has the potential to improve CFPS in general and specifically CFPS-based biosensors (faster response time) while also enabling rapid detoxification/bioremediation through point-of-concern synthesis of similar catalytic enzymes. We further show that such nanoaggregates improve production in diluted CFPS reactions, which can help to save money and extend the amount of these costly reagents. The results are discussed in the context of what may contribute mechanistically to the enhancement and how this can be applied to other CFPS application scenarios.

## Introduction

Cell-free protein synthesis systems (CFPS), also referred to as transcription-translation systems (TX-TL), produce RNA and proteins outside of the confines of a cell using cellular lysate or purified components [1–6]. The products of CFPS can be used as end-products themselves (*e*.*g*., antibodies, enzymes, *etc*.) [2, 7–9] or be used for different applications such as biosensing [1, 10–12], bioremediation [13], or chemical biosynthesis [9, 14]. CFPS offer an alternative to *in vivo* biosynthetic systems with distinct advantages conveyed by their non-living nature. Researchers have shown that CFPS are amenable to the production of proteins that may be inherently toxic or engineered to contain non-natural amino acids or analogues [3, 11, 14–17]. Additionally, CFPS allow users to define reaction conditions enabling reaction enhancement through the elimination of competing metabolic pathways and optimization of select reaction components such as nucleic acid concentrations, for example, to boost reaction efficiency [18, 19]. While CFPS offer a number of advantages over cell-based production platforms they are not without their limitation. CFPS can exhibit lower production rates and efficiencies due to diffusional limitations or absence of cofactor and substrate regeneration systems [2, 4, 5, 20–22]. Moreover, they can have limited shelf life and require long-term storage of critical enzyme components at -80°C.

When implementing CFPS for various applications, two types of systems are typically utilized–crude cell lysate (*e*.*g*., from *Escherichia coli*, yeast, rabbit reticulocytes, insect cells, wheat germ) or a mixture of purified TX-TL components, supplemented with nutrients and other reaction ingredients predetermined to be optimal for a given application [2, 23–28]. The latter can include cofactors, inorganic ions, increased concentrations of certain enzymes, and the like. In general, crude cell lysates are cheaper but can suffer from off-target metabolic pathways that remain present from preparation, uncontrolled proteolytic degradation, or other competing biological processes or inhibitory molecules. Purified TX-TL reactions, in contrast, are typically far more expensive but offer exquisite control over reaction composition [24–27, 29, 30]. The “Protein synthesis Using Recombinant Elements”, or PURE, system is perhaps the most well-known recent embodiment of the latter and is commercially distributed as the PURExpress® *In vitro* Synthesis kit by New England Biolabs with a current cost of ~ US$25 per 25 μL reaction! [6, 25–27, 31]. While the exact composition is now proprietary, it is based on the previously described PUREsystem™ which contained 32 purified protein components in addition to other enzymes, cofactors, *etc*. S1 Table presents an extended list of enzymes and other ingredients, including oligomeric states and concentrations where known or estimated, as drawn from multiple references [25, 32–37]. Critical to our current application, many of these *E*. *coli* TX-TL components are polyhistidine-tagged (His-tagged or His_n_) for ease of recombinant production and purification from cellular expression/production systems [38].

While the PURExpress® system represents the state-of-the-art for purified CFPS, and can produce proteins at yields >100 μg/mL [39], it still suffers from the CFPS disadvantages listed above [5]. For example, the reaction mixture is very dilute (~6.5 g/L protein and ~55 max g/L RNA, see **S1 Table**) [25, 32–37] as compared to *in vivo* conditions (*i*.*e*., 200–300 g/L protein and 75–150 g/L RNA) and therefore may suffer from longer diffusion times and a non-natural, non-molecular-crowding environment [4, 40]. Further, the system produces a fairly high ratio of unfunctional:functional protein, which may be attributed to ribosome stalling causing partially translated products and/or to misfolding as well as other probable reasons that still remain to be elucidated [4, 5, 41]. Addition of chaperones can help increase functional protein yield [4], as can slowing down translation (presumably to allow more time for protein folding) [5, 42]. The system also accumulates inorganic phosphate, which is inhibitory and can further bind and sequester the magnesium needed for translation [5].

Our laboratories have a long-standing interest in the scaffolding of enzymes to nanoparticles (NP, defined herein as < 100 nm in diameter) along with other nanoscale materials such as DNA as a means to enhance enzymatic rates and stability [43–48]. In particular, CdSe/ZnS core/shell quantum dots (QDs) have proven to be an especially advantageous nanomaterial for these purposes as they bind the commonly-used His-tags recombinantly introduced into protein termini for metal affinity-based purification using media displaying Ni^2+^ or other metals chelated into a nitrilotriacetic acid group (NTA). His-tagged proteins and peptides bind to ZnS overcoated QDs almost instantaneously and with nanomolar affinity while further allowing for the oriented immobilization of proteins and enzymes at the NP surface [49]. It has been previously shown that for the special case of enzyme multimers (*e*.*g*., dimers or tetramers), which display multiple pendant His-tags, binding to a QD can form cross-linked nanoaggregated structures which not only can enhance multimeric stability but can also enhance enzyme performance particularly at low enzyme concentrations (*i*.*e*., at or below the dissociation constant) [47]. Enzyme immobilization on QDs is believed to enhance kinetics through multiple mechanisms such as surface effects, which can serve to overcome an enzyme’s rate-limiting step (*e*.*g*., rate of enzyme-product dissociation), by stabilizing enzyme metastructure into a more favorable configuration, and, in the case of enzymatic cascades, providing access to substrate channeling [43–47]. Substrate channeling, or probabilistic channeling, is a phenomenon in which the flux through a given enzymatic cascade is enhanced based on preferential diffusion of intermediates between enzymes rather than to bulk solution [46, 50–55].

**Fig 1:**
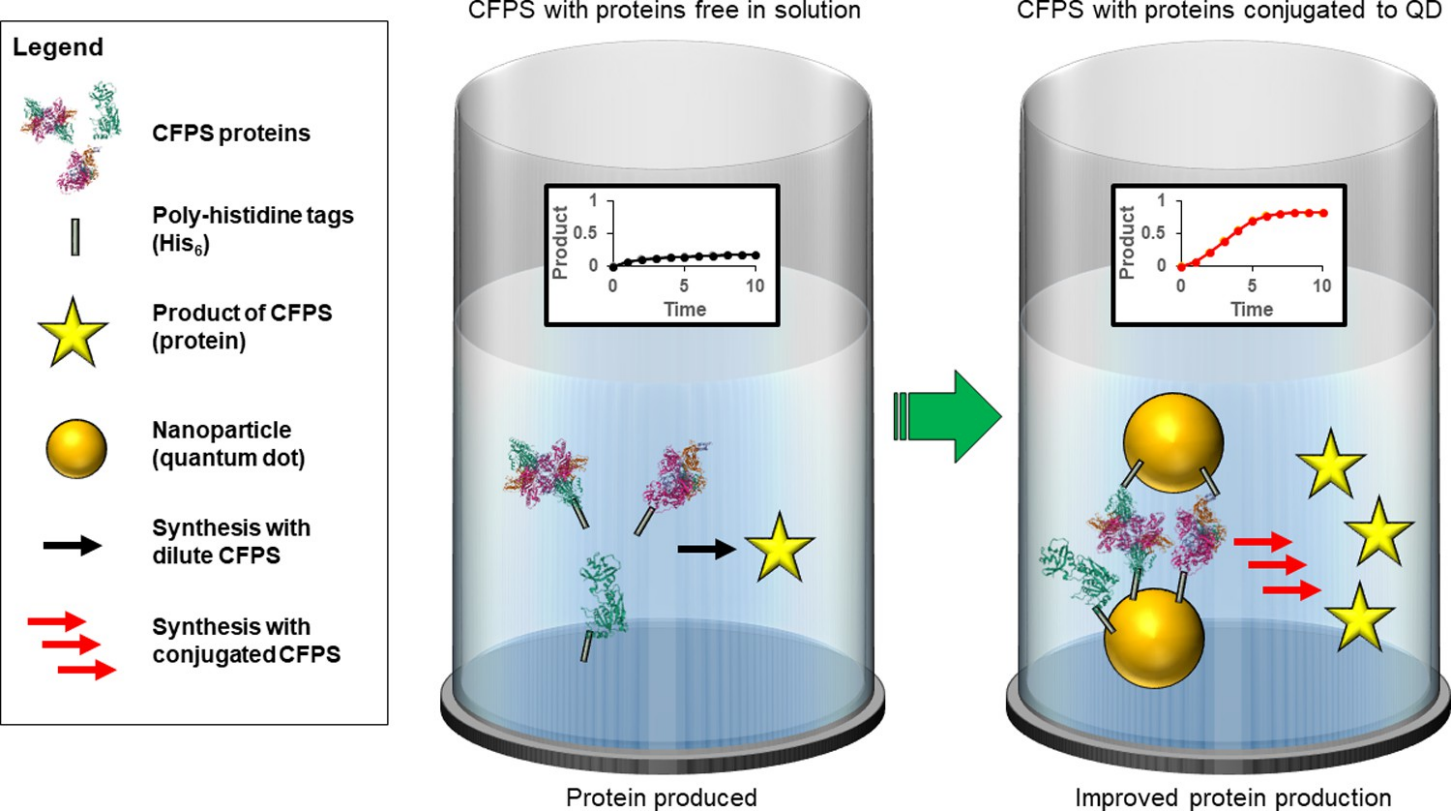
Schematic depicting enhanced cell-free protein synthesis from aggregating the intrinsic enzymes around NPs. (CFPS) systems can suffer from limited reaction rates, likely due to diffusion between components as shown in the reaction to left. CdSe/ZnS core/shell quantum dots (QDs) bind the His-tag of some CFPS components and cross-link into NP-aggregates to bring them into proximity, potentially increasing the catalytic rates or product yield. The enzyme structures shown are known to be present in the CFPS utilized and are represented by structures drawn from the PDB. IDs: 1CRK (mitochondrial creatine kinase), 1FMT (*E*. *coli* methionyl-tRNA(f)Met formyltransferase), and, 3PCO (*E*. *coli* phenylalanine-tRNA synthetase) [56–60].

We reasoned that combining these two technologies—the PURExpress® system and enzyme-QD scaffolding/channeling—could potentially address some of the CFPS challenges listed above by accessing the benefits of the latter, see Fig 1. *A priori*, this would appear challenging since the multi-component PURExpress® system is more complex and far less defined than the previous one or two enzyme-QD systems we had studied [6, 25–27, 32–38, 43–47]. However, some encouragement was found in the work of the Church laboratory, which had previously shown that the addition of bovine serum albumin (BSA) to increase molecular crowding could, in turn, increase protein yield in the PURExpress® system, presumably by bringing the components closer together or optimizing an as yet undefined crowding effect [4]. Using QDs would theoretically do the same but presumably with much less added component (BSA was optimal at 15.5 μM whereas nM concentrations are typically used for QDs due to its multivalency) and by accessing other phenomena such as increased enzyme kinetics due to enzyme immobilization, localized crowding, or increased stability of components as outlined above [4]. This could, in turn, allow for less CFPS material to be needed and therefore decrease cost and/or improve response rates for CFPS-based biosensors.

Herein, we describe proof-of-concept demonstrations of QD-enhanced CFPS by producing two test protein targets: (i) a fluorescent reporter protein for demonstration of potential biosensor applications, and (ii) an organophosphate enzyme to highlight potential downstream detoxification applications. We show that the addition of QDs to PURExpress® can increase fluorescent protein functional yield by up to 20% and that of the enzyme phosphotriesterase by up to 12-fold as compared to equivalent control reactions lacking QD presence. Along with illustrating the potential of this technique, we discuss how this intriguing and potentially useful phenomena could be implemented into other CFPS applications.

## Results

### Assembly of PURExpress® enzymes to QDs and formation of nanoaggregates

**Fig 2:**
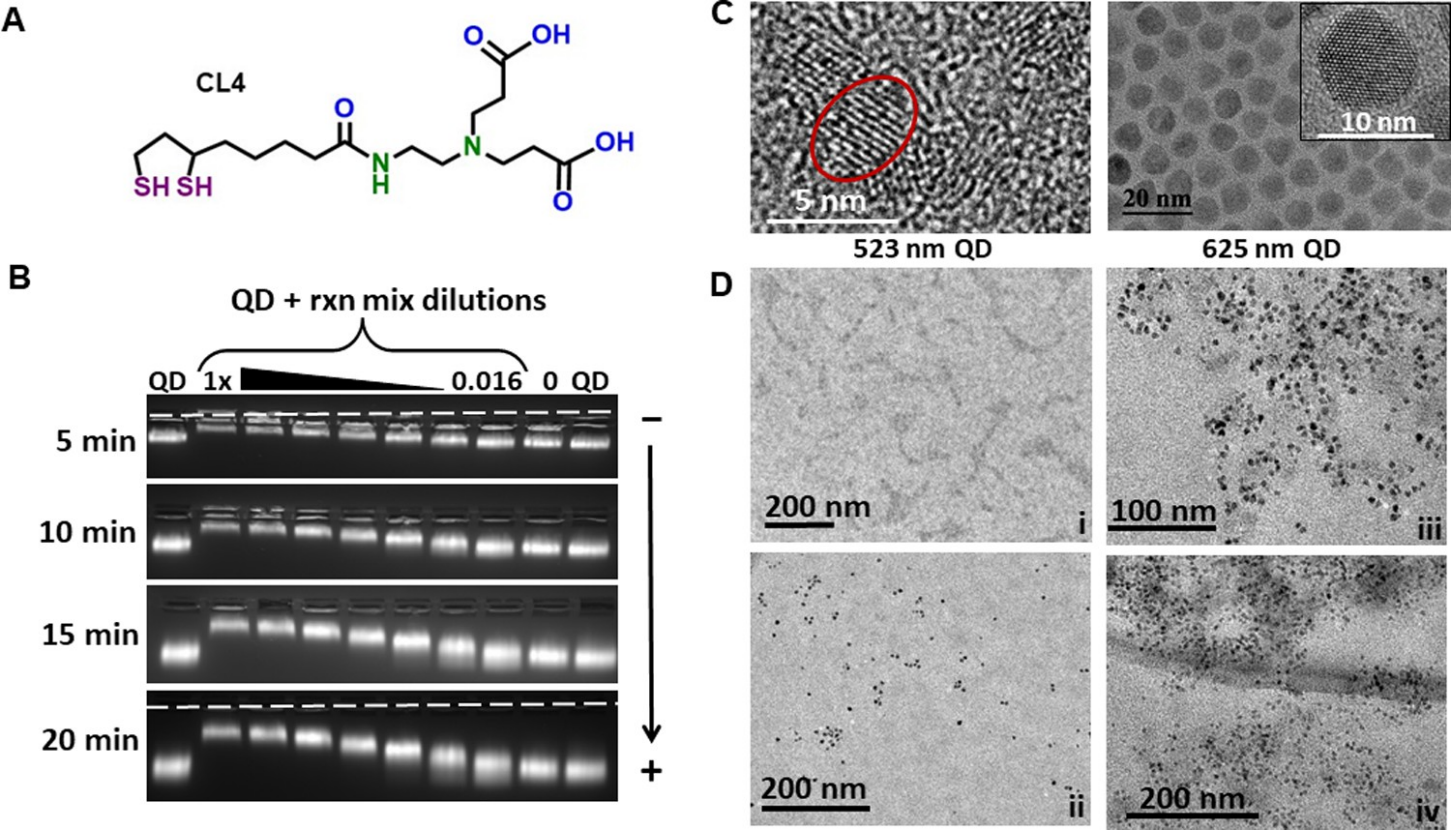
Characterization of PURExpress®–QD conjugates. **(A)** Chemical structure of the CL4 ligand used to make the QDs colloidally stable in aqueous shown in the open dithiol configuration. **(B)** Agarose gel electrophoretic mobility shift assay of 523 nm emitting CdSe/CdS/ZnS core/shell/shell QDs incubated without and with a series of decreasing concentrations of the PURExpress® protein solution. Less mobility is correlated with binding to enzyme and the magnitude of this is decreased as the protein solution is serially diluted. The dashed white line indicates the location of sample wells in the gel. **(C)** Left—High-resolution TEM micrograph of the 523 nm emitting CdSe/CdS/ZnS core/shell/shell QDs with an average diameter of 4.1 ± 0.5 nm. A single QD is circled in red for visualization. Right—High-resolution TEM micrograph of the 625 nm emitting CdSe/ZnS core/shell QDs utilized for nanoaggregation studies due to their larger size and higher electron density which makes for easier imaging. **(D)** TEM micrographs of the PURExpress® protein solution (i), 625 QDs in buffer (ii), and 625 QD mixed with 0.5× PURExpress® solution at two different magnifications (iii, iv). Only when the QDs are mixed with the PURExpress® solution is clustering seen. The grey shading around the QD clusters in (iii, iv) are believed to be the PURExpress® enzymes.

Based on previous work, we selected 523 nm emitting CdSe/CdS/ZnS core/shell/shell QDs with an average diameter of 4.1 ± 0.5 nm for these experiments as these are amongst the smallest size of core/shell type QD material we have available [61–63]. Although not an unequivocal rule, the use of smaller QD materials has been repeatedly correlated with the largest magnitude enhancements in catalytic activity when displaying enzymes on their surface [43, 44, 47, 64]. To provide the QDs with colloidal stability, we utilized the zwitterionic compact ligand (CL4), see Fig 2A for the chemical structure [65]. QDs displaying this surface ligand have been shown to remain stable across a wide range of pH and ion concentrations along with multiple applications in challenging *in vivo* biological environments [66, 67]. More pertinently, the small size of this ligand still allows His_n_-appended proteins to penetrate through and self-assemble to the QD’s Zn-overcoated surface.

Testing of enzyme assembly to the QDs was initially assessed using an agarose gel electrophoretic mobility shift assay (EMSA). Samples containing 5 picomoles of 523 nm QD were mixed with 15 μL of 1× final PURExpress® solution along with a set containing identical volumes and QD concentrations but where the reaction solution underwent 6 serial dilutions by half at every step. This meant that the 7 samples contained from 1× down to 0.016× final PURExpress® solution in the same volume. A negative control of just the same volume of water was set up alongside this series. As shown in Fig 2B, as the ratio of reaction mixture present to QD increased, so too did the gel retention, indicating the QDs were complexing with PURExpress® protein components. We note that the appearance and mobility shifts of the QDs in this gel are almost identical to that observed in many previous assays where assembly of a multitude of other His_n_-displaying proteins and enzymes to QDs was confirmed by EMSA [43, 44, 47, 64, 68]. When the enzymes are multimeric in nature, the multiple His_n_-motifs displayed on opposite ends of the protein can functionally cross-link the QDs into small nanoaggregates as previously revealed by dynamic light scattering (DLS) and transmission electron microscopy (TEM) analysis [47]. Given the multimeric nature of many of the PURExpress® protein components (S1 Table), it was anticipated that such QD crosslinking would also occur here [6, 25–27, 32–38, 43–47]. To test this, a larger size 9.3 nm diameter 625 nm emitting QD was utilized since it is significantly more electron dense than the smaller 4.1 nm diameter 523 nm emitting QDs, see Fig 2C. The latter are almost at the size limit of TEM imaging and the larger QD materials used here for this experiment allow for far easier imaging of cluster formation. Significant clustering of the QDs was indeed confirmed in TEM images when comparing between samples preincubated with 0.5× PURExpress® solution and those that had not; compare Fig 2D micrographs iii and iv *versus* that of ii, respectively.

### Enhancement of active sfGFP production by QD-enzyme nanoaggregates

**Fig 3:**
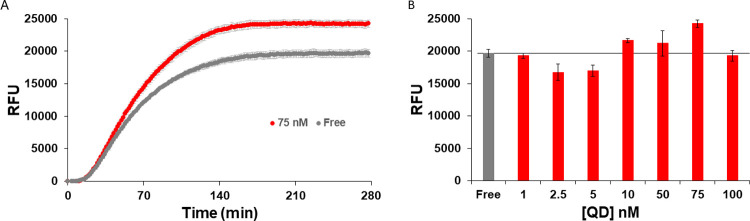
sfGFP production enhancement with QDs. **(A)** Production of sfGFP fluorescence in arbitrary units over time for the optimal 75 nM QD concentration *versus* that of the “free” or QD negative reaction. Samples were excited at 485 nm and fluorescence monitored at 510 nm [69]. Plot for all the QD concentrations can be found in S1 Fig. **(B)** Yield of sfGFP, as estimated by average fluorescence from the end-range of the reactions, over the range of QD concentrations tested (red) and the free reaction (grey). ANOVA *p*-value < 0.05, *F* > *F*_crit_, and Tukey-Kramer analysis, indicated free was statistically different than with QDs for all concentrations except for the 1 nM and 100 nM QDs reactions. The 75 nM QDs condition was significantly different than all other samples (alpha 0.05). For additional information on the statistical analysis, see S2 Fig and S1 Appendix.

Confident that the QDs were forming nanoaggregates with the enzymes present in the PURExpress® solution, we next began investigating the effect of QD presence on PURExpress® protein production. For initial testing, the super-folder green fluorescent protein (sfGFP, ~26.8 kDa) derived from *Aequorea victoria* and appended with a C-terminal Strep tag (Trp-Ser-His-Pro-Gln-Phe-Glu-Lys) was selected due to its previous utilization in cell-free reactions including for biosensing assays [10, 11]. sfGFP has a quantum yield of ~65% and its active fluorophore is estimated to mature in <1 hr [69]. As such, sfGFP fluorescence and any increases/decreases to it in the side-by-side CFPS reactions are amenable to sensitive detection and tracking over time with the Tecan Spark fluorescent microtiterwell plate reader utilized here. Previous reports have noted the importance of the enzyme-QD ratio for enzyme enhancement; therefore, a range of QD concentrations in several increments including 1, 2.5, 5, 10, 50, 75, and 100 nM were added to the PURExpress® reaction and compared to the “free” reaction (no QDs added) [43–47]. The relative amount of fluorescence was then tracked over time and compared. As shown in the representative plots of Fig 3, S1 and S2 Figs, and S1 Appendix, the presence of QDs enhanced several of the reactions compared to the negative control reaction, producing more sfGFP fluorescence. As shown in Fig 3B, a qualitatively biphasic response was noted with the 3 lowest QD concentrations either not affecting sfGFP fluorescence or decreasing it while the next 3 concentrations increased the fluorescence present before returning to baseline for the 100 nM QD concentration. The best performing QD concentration amongst the increments tested was 75 nM, which resulted in an increase of 123% ± 5%. Although only an increase of ca. 20%, statistical testing verified that this was still a significant result in comparison to the fluorescence of the other samples

To probe whether the increases in sfGFP fluorescence was correlated with increased protein production or occurred because of some other mechanism, the same reaction mix was set up with/without 75 nM QD added and samples collected at 0.5, 1, 2, and 6 hours for analysis by Western blotting and total protein staining after polyacrylamide gel electrophoresis (PAGE) separation. The Western blot utilizes an anti-Strep tag antibody enzyme-linked immunosorbent assay (ELISA) approach to detect only fully-formed sfGFP while the visualization of total protein relies on Ponceau S stain to visualize the major protein species. Densitometry analysis of the Western blots reveal that there was no significant change in sfGFP protein production (within error) between the two sample types within the first 120 min, see S3 Fig. This suggests that the addition of QD-enzyme-nanoaggregates access some other mechanism to augment sfGFP fluorescence (*vide infra*).

### Enhancement of active phosphotriesterase production by QD-enzyme nanoaggregates

Given the potential applications of producing enzymes with PURExpress®, whether for biosensors, bioremediation, or chemical production, we next tested whether active enzyme production could be similarly enhanced by QD assembly of the CFPS’s enzymes. We previously have studied the enzyme phosphotriesterase (PTE, EC 3.1.8.1), an organophosphate hydrolase with relevance to decontamination and bioremediation [43, 44, 70–74]. In particular, PTE catalyzes the hydrolysis of organophosphate ester compounds displaying a phosphate center with three surrounding O-linked groups; the latter include commercial pesticides along with structurally similar nerve agents displaying chiral (thio)phosphonate groups such as sarin and tabun. PTE is an obligate dimer with each monomer consisting of a 341 residue unit (~37 kDa, C-terminal Strep-tag) that contains a catalytic binuclear zinc center. The amount of functional PTE produced in this case was measured by assaying its capability to degrade the organophosphate paraoxon, the active metabolite of the insecticide parathion [75], *via* direct monitoring of the absorbance of the resulting *para*-nitrophenol product.

**Fig 4:**
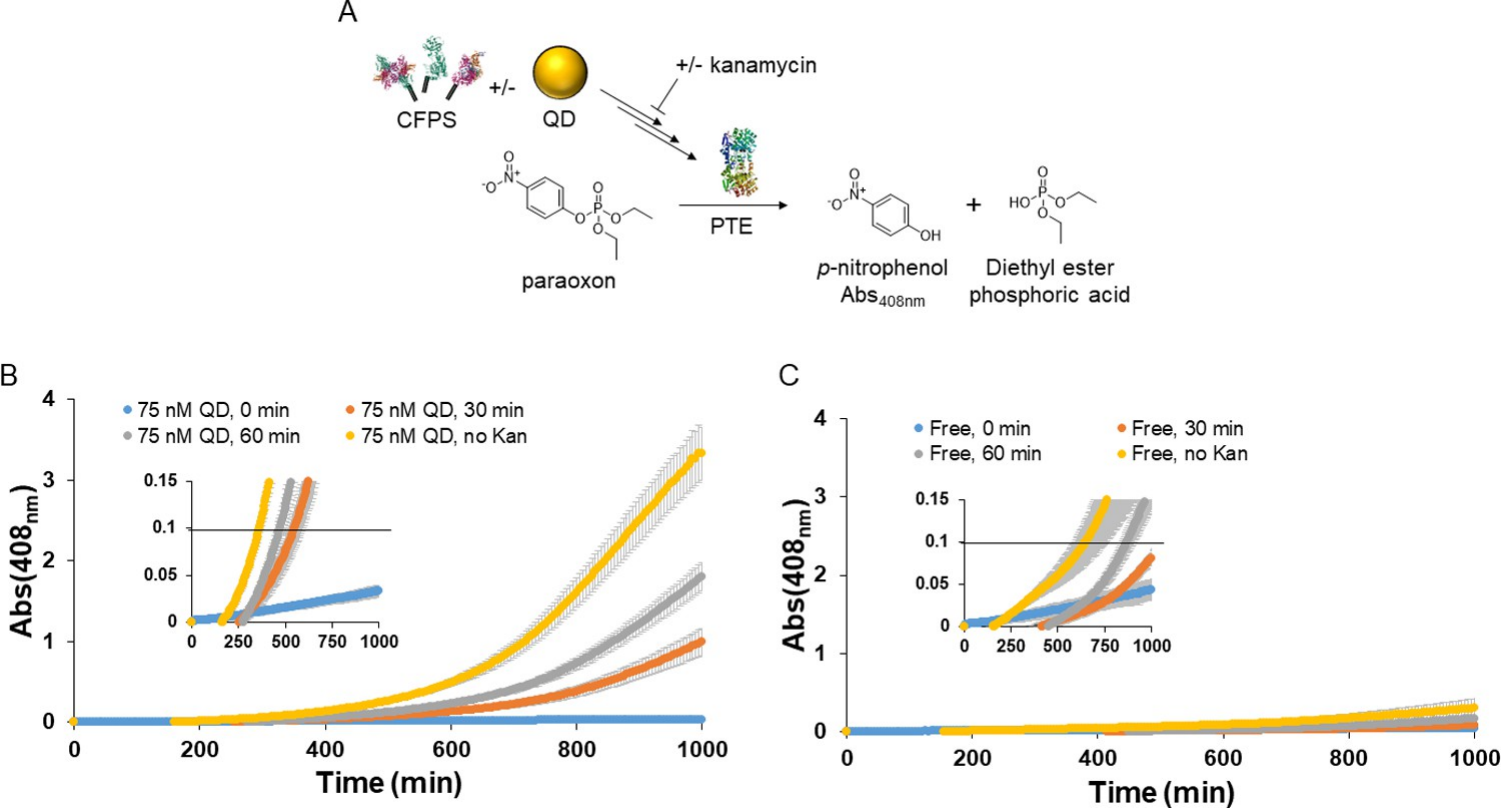
Enhancement of functional PTE production by QDs. **(A)** Reaction setup highlighting stopping of the CFPS reactions with kanamycin at different time points. Paraoxon hydrolysis tracked by measurement of the *p*-nitrophenol absorbance product. Schematic not to scale. **(B)** PURExpress® reaction with QDs produced functional PTE, the activity of which was monitored by absorbance. Kanamycin was added at various time points to quench translation. **(C)** Identical PURExpress® reaction without QDs treated in the same manner as panel (B) produced less functional PTE, resulting in less activity and *p*-nitrophenol product absorbance. PTE PDB ID: IPTA [76]. Other protein structures are the same as shown in Fig 1.

We utilized a somewhat unconventional enzyme assay setup to try to account for both the rate of PTE synthesis and its subsequent enzymatic hydrolysis. In this setup, protein synthesis was quenched at specific time points/intervals to allow for relative quantitation of active enzyme produced by the QD-enhanced PURExpress® reaction *versus* that of controls, see Fig 4A. The antibiotic kanamycin, which inhibits translation, was utilized to stop translation in the CFPS at three separate time-points, namely 0 min, 30 min, and 60 min, in addition to a reaction without quenching. An optimized kanamycin concentration of 20 μg/mL was empirically determined using the sfGFP production reaction (S4 Fig). Then, paraoxon was added to the reactions (final concentration of 2.3 mM) and PTE activity over time monitored by *p*-nitrophenol absorbance at 405 nm (extinction coefficient ∼18,000 M^–1^ cm^–1^). As seen by comparing between Fig 4B (QDs present) to Fig 4C (QD “free” reactions), PTE-mediated hydrolysis of paraoxon was dramatically enhanced with QDs at all time-points, except for the initial 0 min reaction. For the enhanced time-points, *p*-nitrophenol product absorbance was between 10- to 12-fold higher by 1000 minutes with QDs present *versus* that of the free reaction. Interestingly, Western blot analysis again revealed no significant differences in PTE protein concentration between reactions with or without QDs present within the first 120 min, see S5 Fig. To estimate the increased rate for detection in a potential biosensing or bioremediation scenario, the time required to reach a *p*-nitrophenol product absorbance value of ~0.1 was found to be ~4 h faster for the enzyme-QD system (6h *versus* 10 h 40 min, for the non-quenched reactions). It should be noted that as this is only at the proof-of-concept stage, further optimization could potentially decrease the time required to reach a similar absorbance target even further.

### QDs can enhance functional protein production in diluted PURExpress® reactions

**Fig 5:**
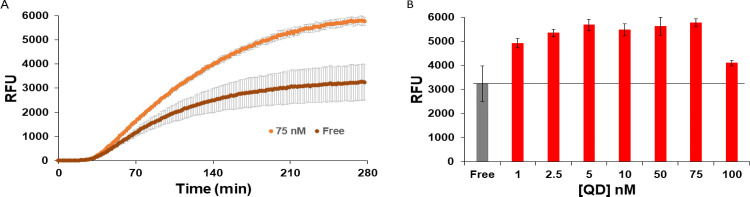
sfGFP production is enhanced with QDs in diluted PURExpress® reaction conditions. **(A)** Production of sfGFP over time with a range of QD concentrations present *versus* a negative control as monitored by fluorescence. Samples were excited at 485 nm and fluorescence monitored at 510 nm [69]. Plot for all the QD concentrations can be found in S6 Fig. **(B)** Yield of functional sfGFP, as estimated by average fluorescence from the end-range of the reactions, over the range of QD concentrations tested (red) as compared to the QD-free reaction (grey). When tested, all samples were statistically different from the free reaction, see S7 Fig and S1 Appendix.

Having shown enhancement of the CFPS with QDs, we next sought to determine if less PURExpress® could be used when enhanced with QDs as a cost/materials savings mechanism. Moreover, any contributions from channeling should become more prominent as the enzymes present are diluted and reactions become more diffusion limited [46, 53–55]. The reactions were diluted in half with nuclease free water and production of functional sfGFP followed by monitoring fluorescence as described above. Similarly, a range of 523 nm QD concentrations including the same 1, 2.5, 5, 10, 50, 75, and 100 nM increments were added to the reactions and tested. Satisfyingly, enhanced production was seen in this diluted regime, with the optimum QD concentration again being 75 nM (Fig 5A). As shown in Fig 5B, S6 and S7 Figs, and S1 Appendix, in the 0.5x PURExpress® reaction, addition of 75 nM QDs again resulted in the largest final fluorescent sfGFP yield as compared to the no-QD control with a *ca*. 80% increase. Moreover, in contrast to the above example, all of the reactions incubated with QDs showed a significant increase in fluorescence produced, none demonstrated any decreases, while the 5, 10, and 50 nM QD-supplemented reactions also yielded essentially the same fluorescence as the 75 nM sample when considering the margin of error. While the addition of QDs to the 0.5× PURExpress® solution did not fully recover the sfGFP fluorescent yield of a 1x solution, giving a yield of 16% (± 4%) without QDs and 29% (± 2%) or one-third with QDs, the increase did provide proof-of-concept that encourages future optimization efforts.

It is important to consider that straight dilution with water to 0.5× reaction conditions means that the reaction buffer, salts, cofactors *etc*., were also all diluted along with the enzymes. Adjusting these to 1× working conditions while allowing the enzymes to remain diluted may be a simple first step towards increasing yield in this scenario. This enhancement correlates well with our previous experience and theory on substrate channeling regarding the benefits of immobilization in low concentration regimes [46]. Note that a further dilution of PURExpress (0.1x) was inconclusive due to the low signal intensity of sfGFP, as shown in S8 Fig.

## Discussion and conclusions

Herein, a proof-of-concept study demonstrated that addition of QDs could enhance functional protein and enzyme production in PURExpress® CFPS reactions. Production of sfGFP was initially increased ~20% with the addition of QDs. Production of PTE was also increased, with the final product signal up to 12-fold higher and a (arbitrarily imposed) signal detection threshold of 0.1 being reached over 4 h faster with the addition of QDs. Both of these enhancements could be beneficial for CFPS-based biosensors (*i*.*e*., sfGFP readouts or organophosphate detection), especially where a more rapid response is desired. This benefit could also extend to bioremediation efforts or other enzymatic applications (*e*.*g*., small molecule synthesis). Further, the ability to enhance production in diluted PURExpress® reactions portends well to reduce costs and materials required for such assays. In an era of ever diminishing research funding, a base cost of ~ US$25 per reaction is not trivial [31]. We acknowledge that the additional cost of commercial QDs may not appear economical at first glance, but it is worth exploring this point in the context of the current scenario. Commercial QDs of a similar size as those used here are available surface-functionalized with carboxylated ligands; it has been shown that these carboxyl groups can functionally mimic an NTA moiety by chelating Ni^2+^ and allow for (His)_n_-appended proteins to coordinate to their surface in a similar manner as described above [68, 77]. Such commercial QDs are currently priced at US$470 for 2 μMoles [78]. By using half of a single PURExpress® reaction (~$12.50) and supplementing the reaction with the equivalent of 5 nanoMolar QD, as in the sfGFP experiment above, the QD portion of the cost for augmenting a single reaction is negligible at $0.001 and only increases to $0.02 for the higher 75 nM QD concentration. This added cost does not even reach a threshold of 1% of the PURExpress® portion of the reaction. Of course, we utilize QDs here merely as a model NP platform that displays the requisite colloidal stability and surface chemistry, and anticipate that these advantages may transfer to differently tagged TX-TL components and/or alternative NP platforms, which may be even cheaper.

Discerning the mechanism behind QD enhancements in the PURExpress® reaction is beyond the scope of this study. Although an increase in functional product activity for samples with QDs present *versus* those without, including when sample concentration is diluted, is potentially indicative of a channeling contribution [46, 53–55], the lack of a clearly visible early difference between any of the protein product concentrations suggest this not to be that simple an underlying cause in this case. We hypothesize that the observed increases are partially due to improving the functional:unfunctional protein ratio. As mentioned above, this is an issue with the PURExpress® system discussed in previous publications where the yield of functional protein was lower than the yield of total protein [4, 5, 41]. In line with this, PAGE separation followed by Western blotting and densitometry measurements of sfGFP product in reaction samples indicated that at up to 120 minutes, there was no enhancement in total sfGFP produced by QD addition, yet an enhancement in fluorescence was seen by this time (Fig 3A and S3 Fig). This suggests that at least some of the fluorescence enhancement is due to an analogous increased ratio of functional:unfunctional protein being produced. A similar trend is seen with PTE as functional PTE is enhanced during the beginning of the PURExpress® reaction when supplemented by QDs, but not total PTE (Fig 4B, 4C, and S5 Fig). How QDs are increasing functional protein yield is not readily clear; we are not aware of any protein folding or chaperone-like function associated with QDs, though we cannot rule it out. It is difficult to discern whether translation is slowing down (allowing more time for functional folding); for sfGFP, total protein production was perhaps slowed at 30 minutes (S3 Fig). No differences were obvious regarding lower molecular weight bands in the total protein stained gels for either sfGFP or PTE that could indicate truncated translation products (the anti-Strep tag was on the C-terminus, precluding finding truncated translation products in the Western blots). We also note that for sfGFP, total protein production was increased between 120–360 minutes whereas fluorescence was not, perhaps indicating that this protein was non-functional (*e*.*g*., misfolded) or there are other extraneous circumstances (*e*.*g*., limited O_2_ available for sfGFP maturation) [69]. We discount enhancement of PTE activity by direct binding to the QD surface, as previously shown with QDs and gold NPs [43, 44], as a contributor to improved function since the reaction mixtures enzymes’ (His)_n_ bind first and, even if there were some available QD surface binding sites left, the protein products examined here all lack the requisite (His)_n_ within their sequence.

There are other aspects to consider in relation to QD enhancement of PURExpress® reactions. One is that increased molecular crowding through use of BSA supplementation has been shown to enhance PURExpress® reactions previously [4]. In that work, 15.5 μM BSA (66.5 kDa, ~8 nm diameter) [79] was an optimal concentration. Considering that those concentrations/mass of BSA would likely displace a much greater volume compared to the 75 nM QD used here (diameter ~4.1 nm) [43], it is unlikely that the QDs are causing molecular crowding by simple volumetric/displacement properties. For more information on molecular crowding, including aspects to cell-free systems, please see references Tan *et al*. and Rustad *et al*. [80, 81] If the same general mechanism is responsible, it is likely due to the direct nanoconjugation of QDs to PURExpress® components bringing them into close proximity within larger nanoaggregates, as reflected in the TEM images. Other potential contributory mechanisms for the observed QD enhancement of PURExpress® reactions could include increased stabilization of multimeric enzymes, increased activity of individual immobilized PURExpress® enzyme components and/or substrate/probabilistic channeling between them, potential sequestration of inhibitory compounds or localized enrichment of a key substrate, along with other unknown mechanisms. We do note that there is evidence supporting the notion that QD binding or display stabilizes the intact structure and subsequent function of multimeric enzymes and especially at low concentrations [47]. Despite these unknowns, we anticipate that further research into NP enhancement of such CFPS could potentially still be quite useful as it may reveal how to further optimize productivity and efficiency in these reactions along with providing insight into how to accomplish the same in other complex multicomponent (although still nanoscale) enzymatic and biosynthetic environments.

## Materials and methods

### Reagents

Paraoxon, buffers, salts, and other reagents were purchased from Sigma-Aldrich (St. Louis, MO, USA). PURExpress® *In Vitro* Protein Synthesis kits, DNA purification kits, and high efficiency DH10beta competent cells were purchased from New England Biolabs (NEB, Ipswich, MA, USA). Precast Mini-PROTEAN TGX 4–15% gradient gels were purchased from Bio-Rad (Hercules, CA, USA). Anti-streptavidin-alkaline phosphatase conjugate was purchased from Jackson ImmunoResearch Laboratories, Inc. and 1-Step NBT/BCIP precipitating substrate were purchased from ThermoFisher Scientific (Waltham, MA, USA). DNA amplification was accomplished using Q5 High-Fidelity DNA polymerase (NEB). Black 384-well microtiter non-treated polystyrene plates with flat, clear bottoms were from Corning (Corning Inc., NY, USA).

### Genetic constructs

Genes encoding for superfolder GFP (sfGFP) [82] and phosphotriesterase from *Brevundimonas diminuta*, (PTE, EC 3.1.8.1) [71] were cloned into pY71 plasmid by *in vivo* assembly following a previously described protocol [83]. A strep tag was appended at the C-terminus of the genes and the final constructs pY71-sfGFP and pY71-PTE were confirmed by sequencing. Plasmids were purified from *E*. *coli* DH10beta cells using the Plasmid Miniprep Kit (NEB). The final DNA was eluted using molecular biology grade water. A working concentration of 50 ng/μl was used, and the DNA was stored at -20°C.

### CFPS reaction and QD addition

FPS reactions were performed with the PURExpress *in vitro* protein synthesis kit. 25 μL of the reaction contained 10 μL Solution A, 7.5 μL Solution B, and nuclease-free water added to reach the final volume. Reactions at 0.5x strength had ½ volume of Solutions A and B, and reactions at 0.1x strength had 1/10 volume of Solutions A and B; nuclease-free water was added to reach the final volume of 25 μL. For designated reactions, QDs were then added and allowed to assemble to reaction components for 30 min on ice. QDs were CdSe/CdS/ZnS core/shell/shell QDs with emission maxima centered at ∼523 nm and solubilized with dihydrolipoic acid-zwitterionic compact ligand (DHLA-CL4 or CL4) as described [61–63, 65]. After assembly, 20 μL of PURExpress® reaction with or without QDs was added to the wells of a 384 well microtiter plate followed by addition of 5 μL DNA solution in water (100 ng DNA of pY71-sfGFP or pY71-PTE). Reactions for immunoblot were carried out in tubes at 37°C and stopped at different time points by 4x Laemmli sample buffer containing 5mM β-mercaptoethanol (βMe).

### sfGFP assay

For sfGFP, fluorescence kinetics were measured at an excitation wavelength of 485 nm and an emission wavelength of 510 nm in a plate reader at 37°C for the indicated time. Here, protein synthesis and chromophore maturation is reported in relative fluorescence units (RFU) which is the measured value subtracted from the background fluorescence of the reaction components; the latter is defined as fluorescence intensity at the initial time value.

### PTE enzyme assay

For CFPS reactions with QDs, QDs were added and allowed to assemble to reaction components for 30 min on ice. Then CFPS reactions were run at 37 ⁰C until quenched with 20 μg/mL kanamycin at time points of 0, 30, and 60 min, with another CFPS reaction run without quenching. Following the 60 min quench, PTE activity was assayed using paraoxon (diethyl 4-nitrophenyl phosphate) as a substrate. Paraoxon was first diluted 1:1,000 from a neat concentration of ~4.629 M in 2-(Cyclohexylamino)ethanesulfonic acid (CHES) buffer (50 mM, pH 8) in the presence of 10 μM cobalt chloride for a concentration of 4.629 mM. The reactions and plasmid DNA (25 μL) and 25 μL of paraoxon solution (1:1000 dilution in CHES buffer) were assembled in the wells of a 384 well black microtiter plate for a final paraoxon concentration of 2.3 mM. Enzyme-mediated hydrolysis of the paraoxon substrate to *p*-nitrophenol was monitored at 408 nm, and reported absorbance is the value with the initial time 0 value subtracted.

### SDS-PAGE and Western blot analysis

The cell free reactions were terminated by addition of Laemmli sample buffer containing βMe and run on a gradient (4–15%) SDS-PAGE gel in Tris-glycine running buffer under reducing conditions at 150 V for 60 min then transferred to a nitrocellulose membrane at 15 V for 15 min in 10% methanol transfer buffer. The molecular weight marker used was Precision Plus Protein Dual Color Standards (Bio-Rad, Hercules, CA, USA). The membrane was stained using Ponceau S and imaged using a Bio-Rad ChemiDoc imager, then blocked in 3% skimmed milk in PBS-T (PBS with 0.05% Tween-20), and probed with 1:5000 dilution of streptavidin-alkaline phosphatase conjugate for 1 hr at room temperature. The membrane was developed using a chromogenic alkaline phosphatase NBT (nitro-blue tetrazolium chloride) and BCIP (5-bromo-4-chloro-3’-indolyphosphate p-toluidine salt) substrate solution and imaged using Bio-Rad ChemiDoc imager.

### Densitometry

Bands were quantified with ImageJ, a Java-based image analysis package widely used for measurement of peak intensity of the band of the expected molecular weight [84]. Three independent Western blots were used for quantification and the results were plotted as average intensity ± standard error.

### Transmission electron microscopy

A 10 μL aliquot of PURExpress Solution A was mixed with 7.5 μL of Solution B, H_2_O, and assembled and 75 nM QD (625 nm emission maxima) or water (control) at 4°C for 30 min. From the assembled reaction a 5–10 μL volume of the sample was spread onto ultrathin carbon/holey support film on a 300 mesh Au grid (Ted Pella, Inc.) and left to dry at room temperature. The samples were characterized using a JEOL 2200-FX analytical high-resolution transmission electron microscope (TEM) with a 200 kV accelerating voltage [47, 85].

## Supporting information

S1 Fig sfGFP production enhancement with full range of QD concentrations tested at 1x PURExpress® reaction conditions. **(A)** Production of sfGFP fluorescence in arbitrary units over time *versus* that of the “free” or QD negative reaction. Samples were excited at 485 nm and fluorescence monitored at 510 nm [69].
(TIF)

S2 Fig Additional statistics of sfGFF production at 1x PURExpress® reaction conditions. ANOVA *p*-value was < 0.05 and *F* was > *F*_crit_, indicating significant difference between treatments. Tukey-Kramer analysis was then done. Stars indicate treatments were significantly different from each other (alpha 0.05).
(TIF)

S3 Fig Representative SDS-PAGE and Western blot of sfGFP production. (A) Total sfGFP produced over time by densitometry analysis of Western blot. Note this presumably includes both initially functional and unfunctional protein. (B) Representative SDS-PAGE stained with Ponceau S (top) and Western blot probed with a streptavidin-alkaline phosphatase conjugate (bottom) used for densitometry analysis. Note the lack of significant bands indicating truncated sfGFP products in the top images.
(TIF)

S4 Fig Inhibition of cell-free reaction by kanamycin. Indicated concentration of kanamycin was added after 30 min in cell-free reaction and change in sfGFP fluorescence was monitored for 90 min. Initial time point is the time of kanamycin addition.
(TIF)

S5 Fig Representative SDS-PAGE and Western blot of PTE production. (A) Total PTE produced over time by densitometry analysis of Western blot. Note this presumably includes both initially functional and unfunctional protein. (B) Representative SDS-PAGE stained with Ponceau S (top) and Western blot probed with a streptavidin-alkaline phosphatase conjugate (bottom) used for densitometry analysis. Note the lack of significant bands indicating truncated PTE products in the top images.
(TIF)

S6 Fig sfGFP production enhancement with full range of QD concentrations tested at 0.5x PURExpress® reaction conditions. **(A)** Production of sfGFP fluorescence in arbitrary units over time *versus* that of the “free” or QD negative reaction. Samples were excited at 485 nm and fluorescence monitored at 510 nm [69].
(TIF)

S7 Fig Additional statistics of sfGFF production at 0.5x PURExpress® reaction conditions. ANOVA *p*-value was < 0.05 and *F* was > *F*_crit_, indicating significant difference between treatments. Tukey-Kramer analysis was then done. Stars indicate treatments were significantly different from each other (alpha 0.05).
(TIF)

S8 Fig sfGFP production enhancement with full range of QD concentrations tested at 0.1x PURExpress® reaction conditions. **(A)** Production of sfGFP fluorescence in arbitrary units over time *versus* that of the “free” or QD negative reaction. Samples were excited at 485 nm and fluorescence monitored at 510 nm [69].
(TIF)

S1 Table Estimated characteristics of PURExpress® components. (XLSX)

S1 Appendix Method for additional statistical analysis of sfGFP production. (DOCX)

S1 Raw image Raw images of all gels and blots. (TIF)

S1 Raw data Data underlying the findings in this manuscript. (ZIP)
