# Supplementary figures and images for: Self-assembled nanoparticle-enzyme aggregates enhance functional protein production in pure transcription-translation systems
Source: PLoS One. 2022 Mar 17;17(3):e0265274. doi: 10.1371/journal.pone.0265274 (PMC8929567; doi:10.1371/journal.pone.0265274)

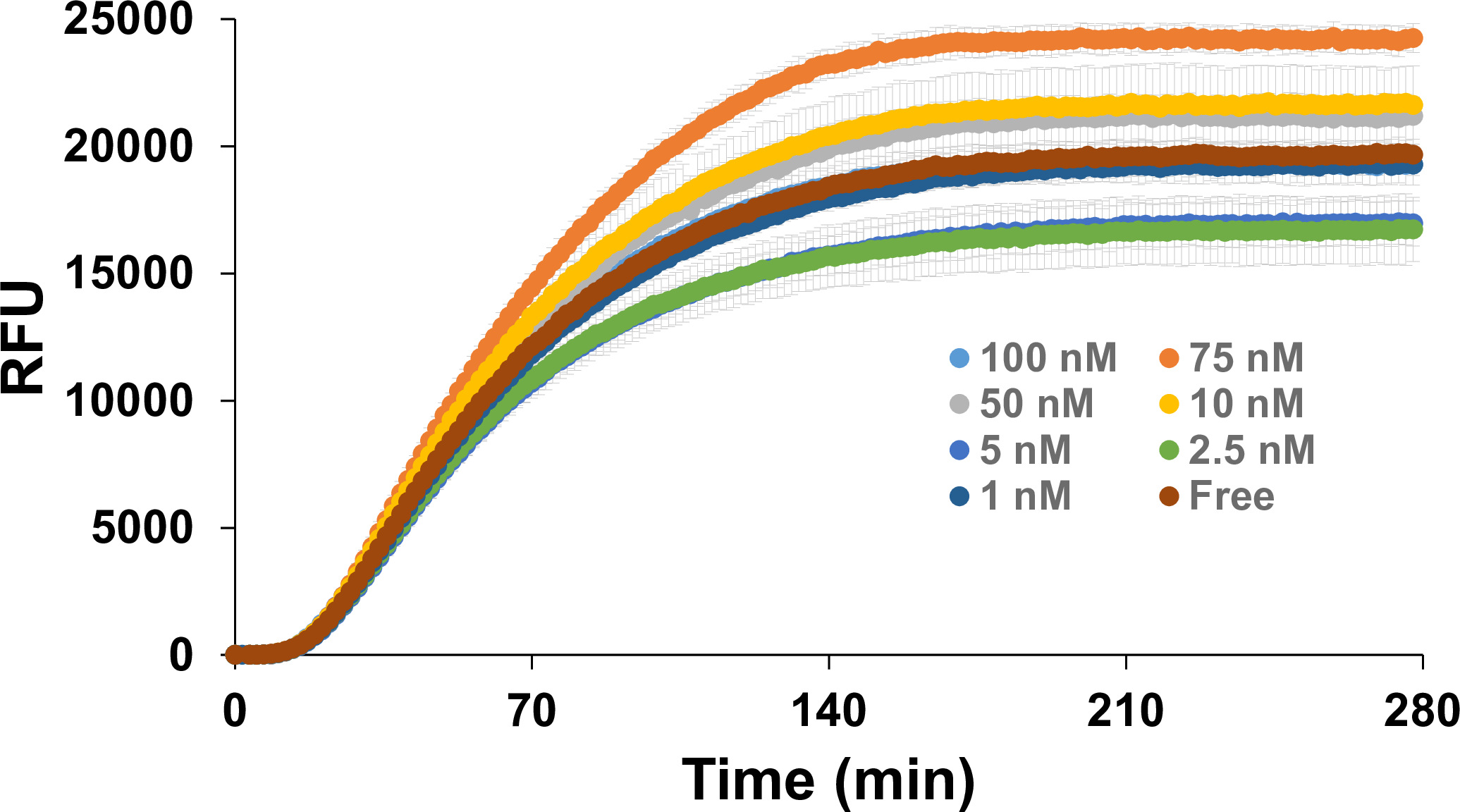

Supplement: S1 Fig — (A) Production of sfGFP fluorescence in arbitrary units over time versus that of the “free” or QD negative reaction. Samples were excited at 485 nm and fluorescence monitored at 510 nm [69]. (TIF) [file pone.0265274.s001.tif]

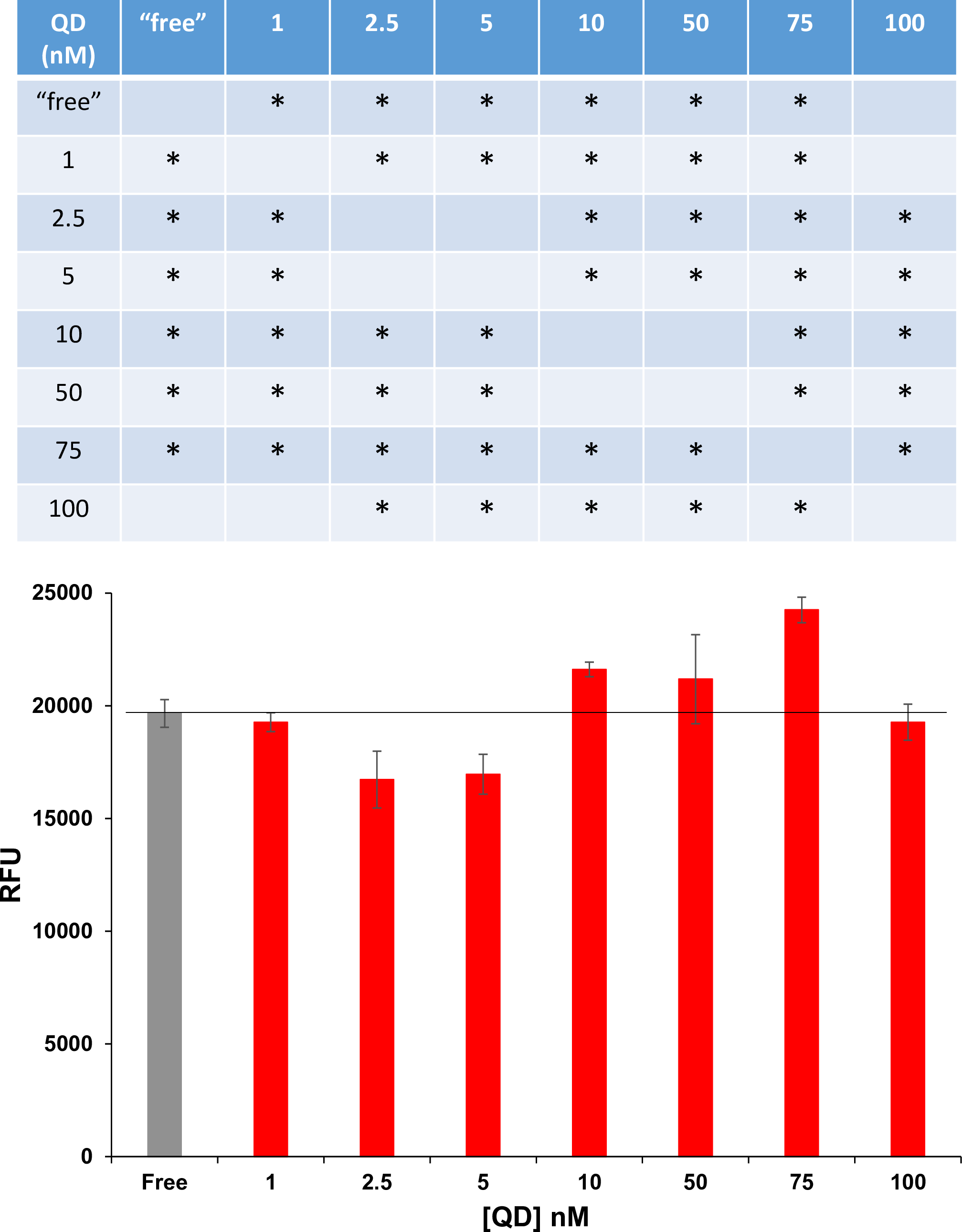

Supplement: S2 Fig — ANOVA p-value was < 0.05 and F was > Fcrit, indicating significant difference between treatments. Tukey-Kramer analysis was then done. Stars indicate treatments were significantly different from each other (alpha 0.05). (TIF) [file pone.0265274.s002.tif]

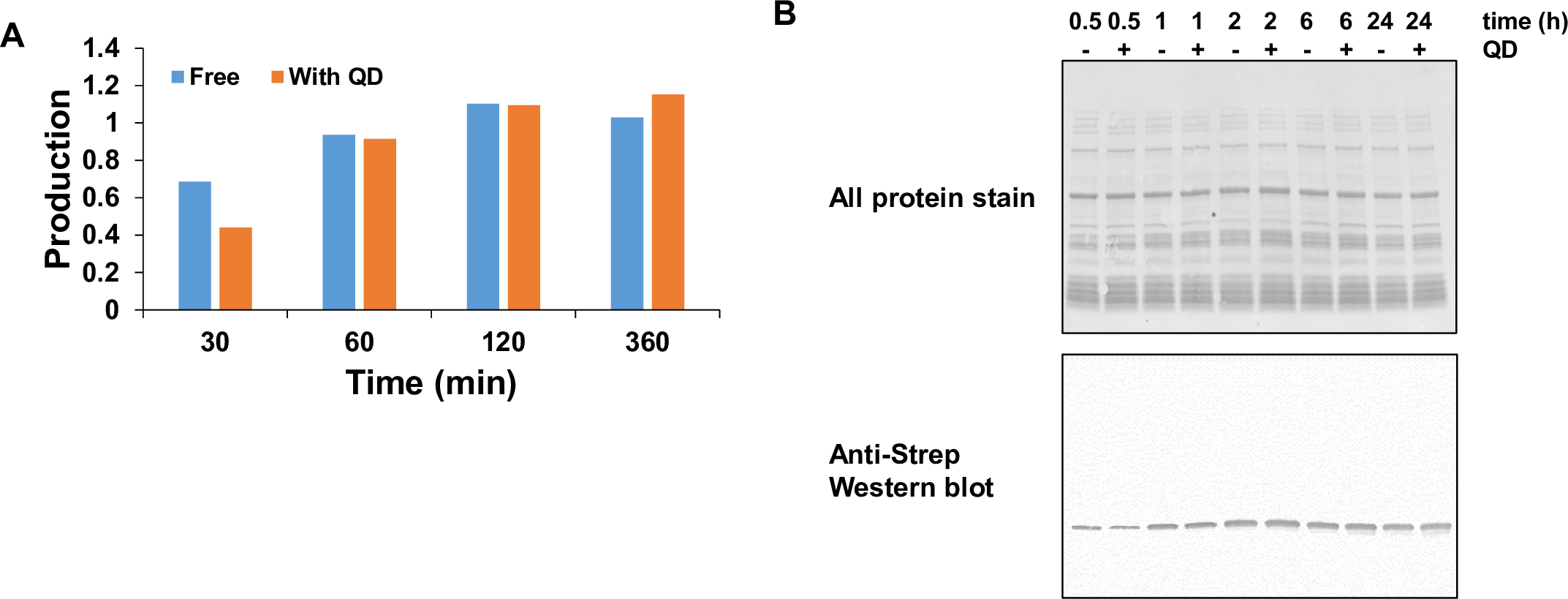

Supplement: S3 Fig — (A) Total sfGFP produced over time by densitometry analysis of Western blot. Note this presumably includes both initially functional and unfunctional protein. (B) Representative SDS-PAGE stained with Ponceau S (top) and Western blot probed with a streptavidin-alkaline phosphatase conjugate (bottom) used for densitometry analysis. Note the lack of significant bands indicating truncated sfGFP products in the top images. (TIF) [file pone.0265274.s003.tif]

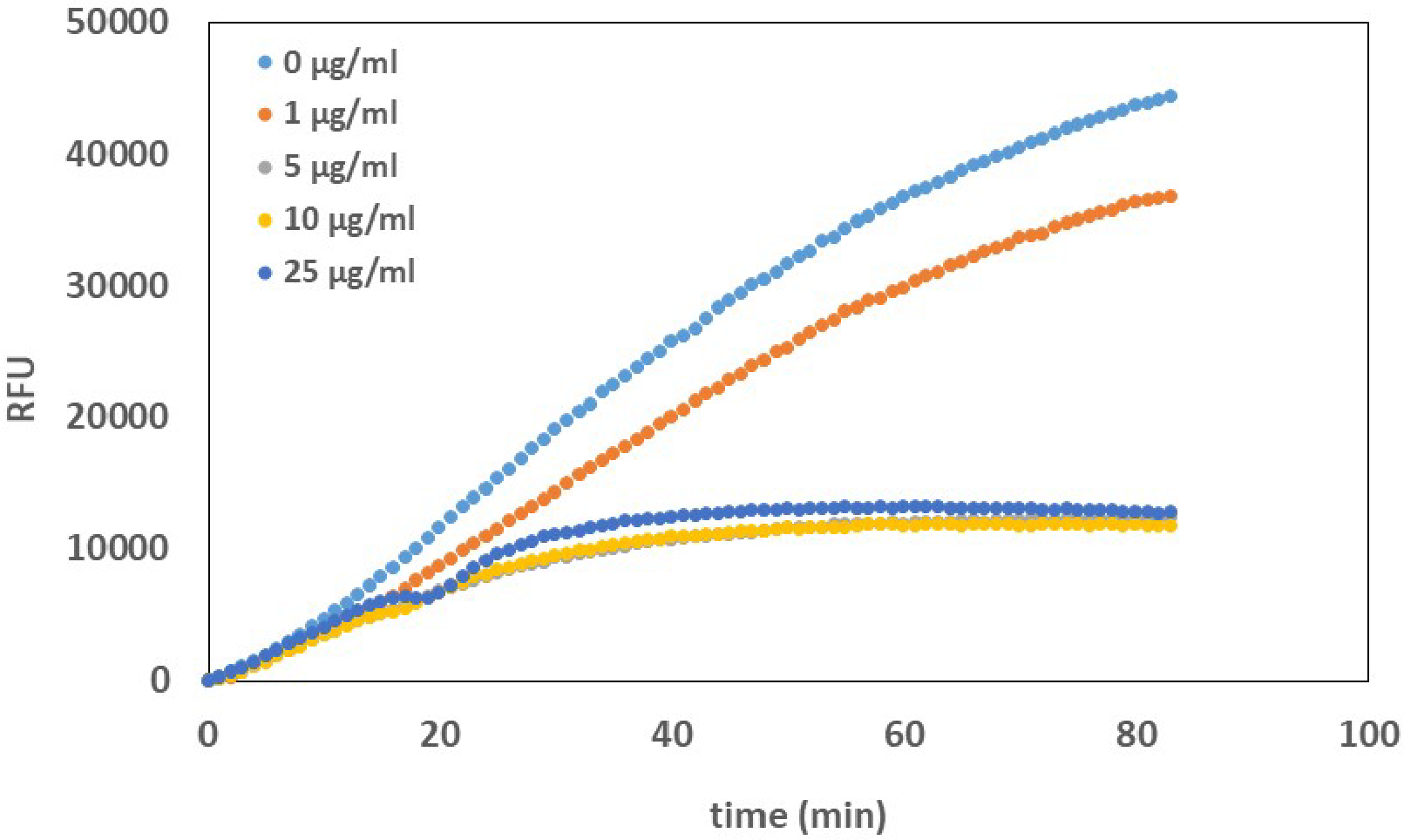

Supplement: S4 Fig — Indicated concentration of kanamycin was added after 30 min in cell-free reaction and change in sfGFP fluorescence was monitored for 90 min. Initial time point is the time of kanamycin addition. (TIF) [file pone.0265274.s004.tif]

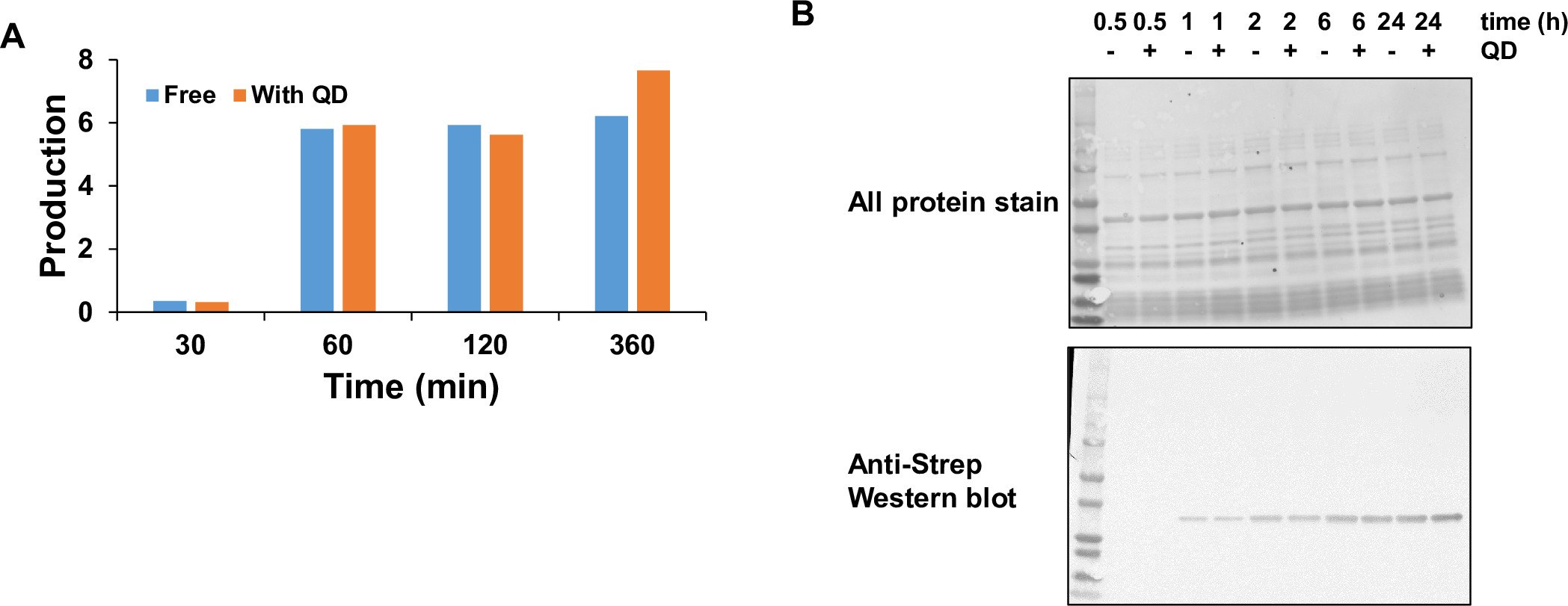

Supplement: S5 Fig — (A) Total PTE produced over time by densitometry analysis of Western blot. Note this presumably includes both initially functional and unfunctional protein. (B) Representative SDS-PAGE stained with Ponceau S (top) and Western blot probed with a streptavidin-alkaline phosphatase conjugate (bottom) used for densitometry analysis. Note the lack of significant bands indicating truncated PTE products in the top images. (TIF) [file pone.0265274.s005.tif]

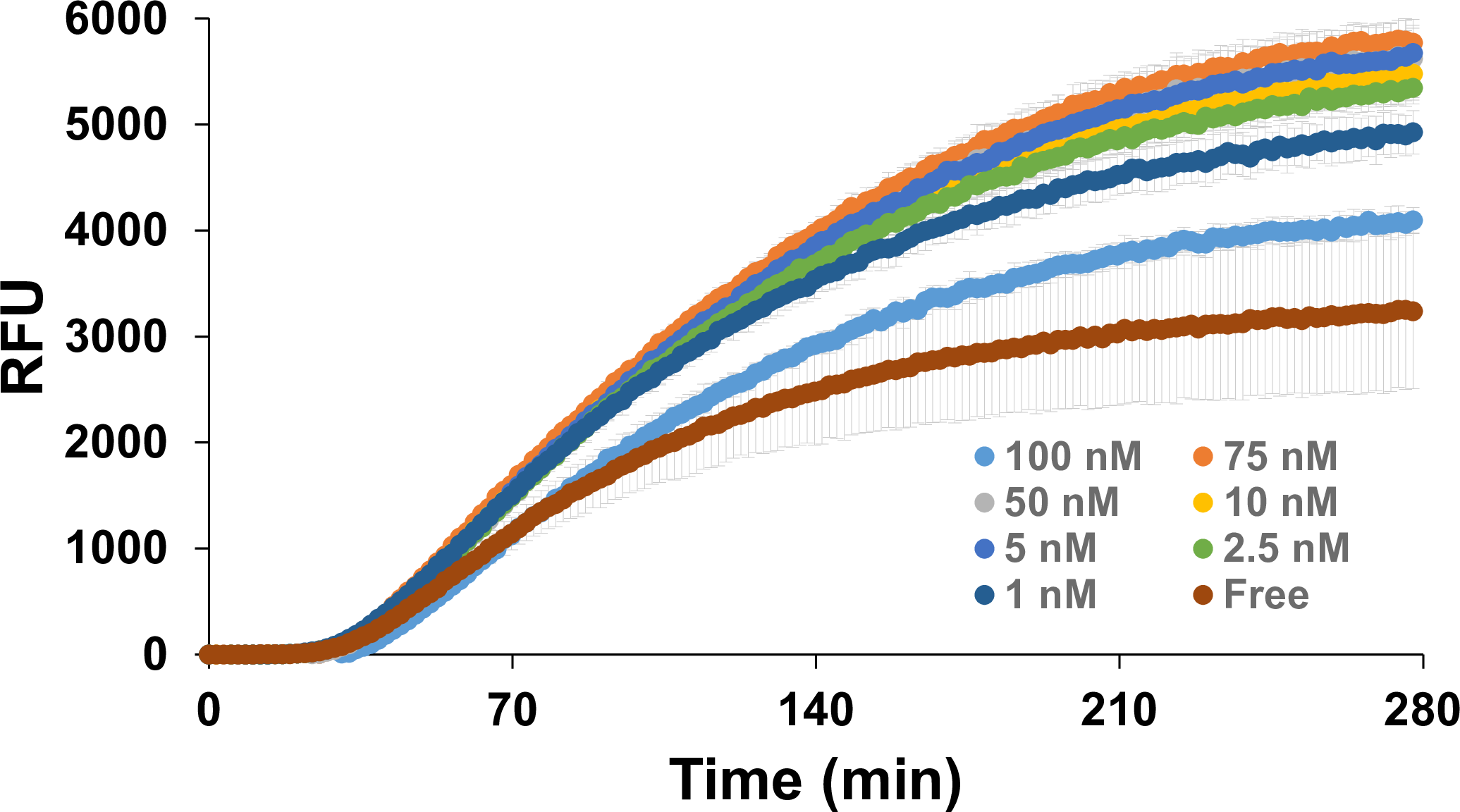

Supplement: S6 Fig — (A) Production of sfGFP fluorescence in arbitrary units over time versus that of the “free” or QD negative reaction. Samples were excited at 485 nm and fluorescence monitored at 510 nm [69]. (TIF) [file pone.0265274.s006.tif]

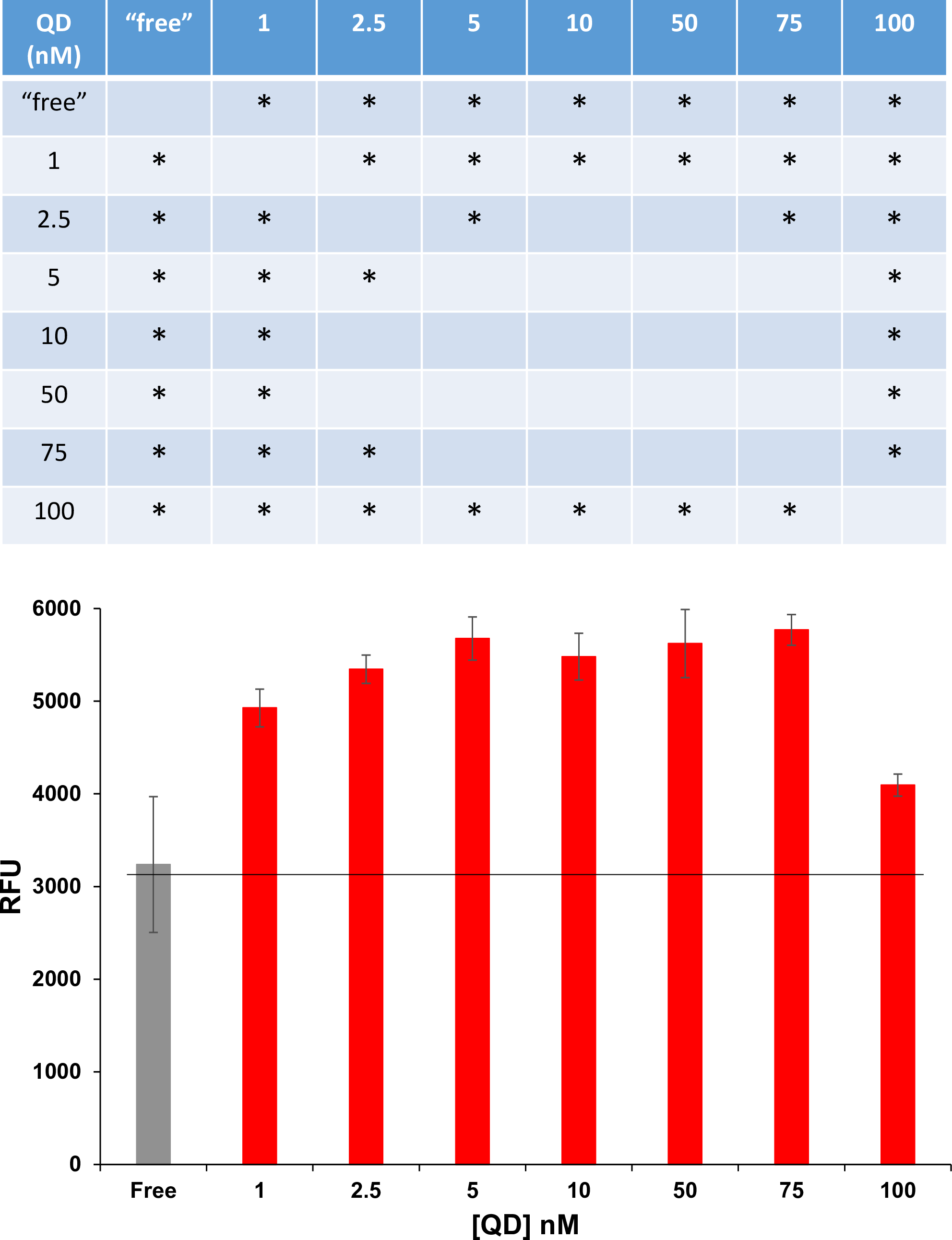

Supplement: S7 Fig — ANOVA p-value was < 0.05 and F was > Fcrit, indicating significant difference between treatments. Tukey-Kramer analysis was then done. Stars indicate treatments were significantly different from each other (alpha 0.05). (TIF) [file pone.0265274.s007.tif]

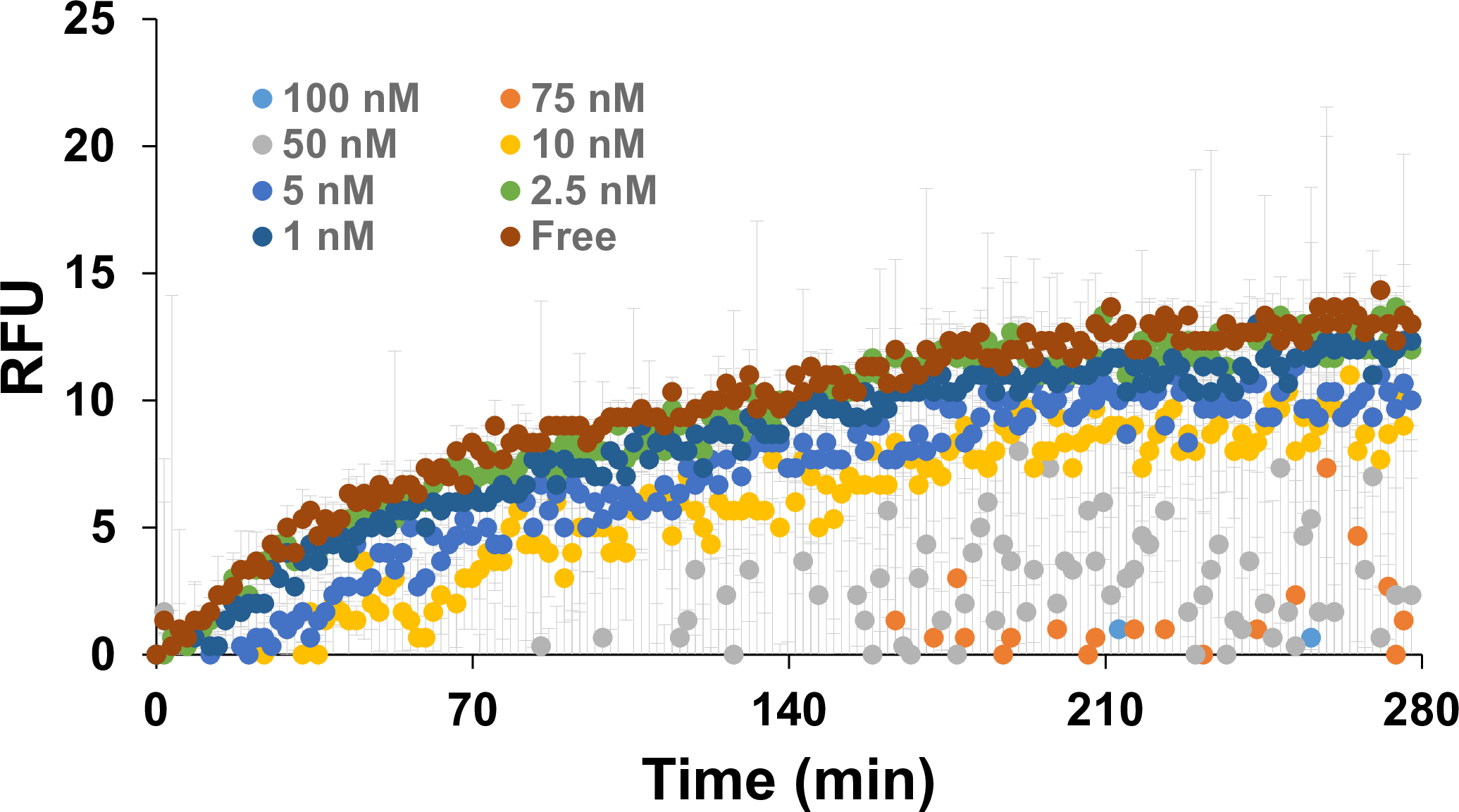

Supplement: S8 Fig — (A) Production of sfGFP fluorescence in arbitrary units over time versus that of the “free” or QD negative reaction. Samples were excited at 485 nm and fluorescence monitored at 510 nm [69]. (TIF) [file pone.0265274.s008.tif]

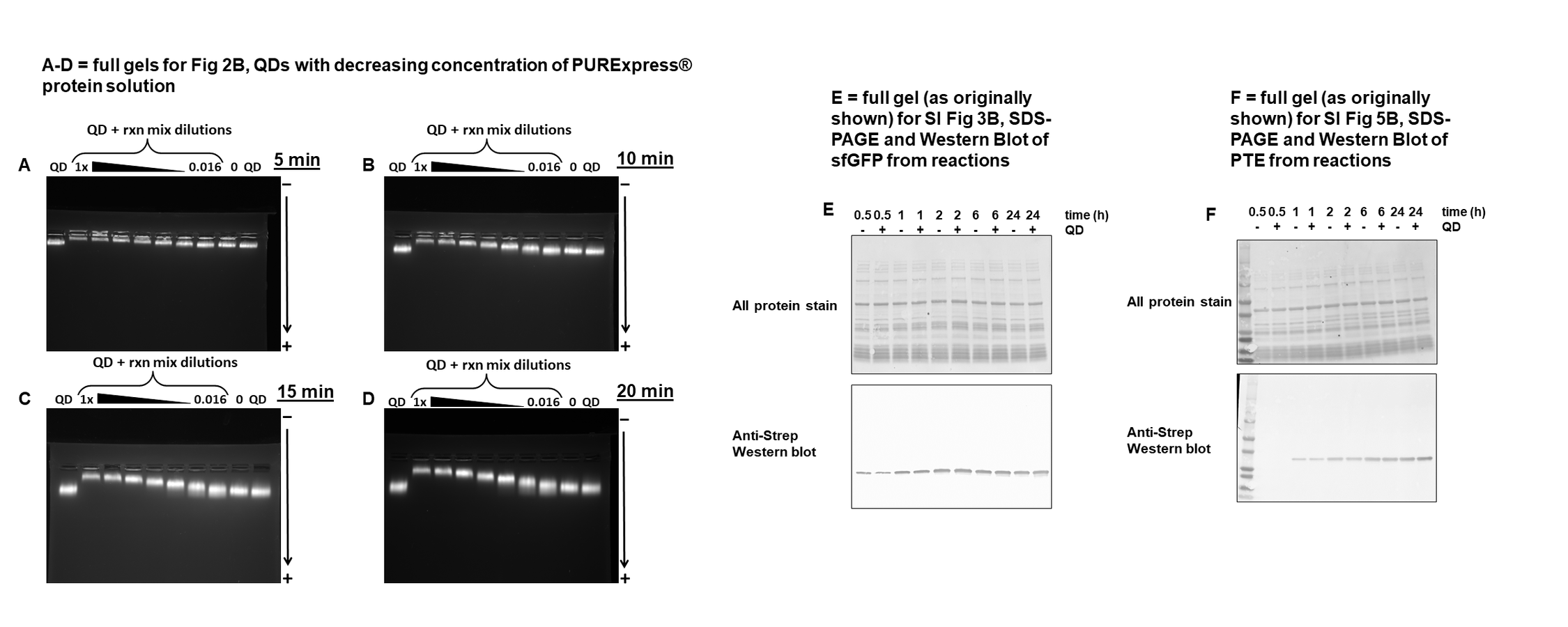

Supplement: S1 Raw image — (TIF) [file pone.0265274.s011.tif]
