# Supplementary material for: Self-assembled nanoparticle-enzyme aggregates enhance functional protein production in pure transcription-translation systems
Source: PLoS One. 2022 Mar 17;17(3):e0265274. doi: 10.1371/journal.pone.0265274 (PMC8929567; doi:10.1371/journal.pone.0265274)
Supplement: S1 Appendix — (DOCX) [file pone.0265274.s010.docx]

**Statistical analysis**

For statistical significance determination of final sfGFP production using the 1x and 0.5x reaction with different concentrations of QDs and the free reaction, first a single factor ANOVA was conducted using Excel 2016 Data Analysis (Microsoft, Redmond, WA, USA) to determine if the *p*-value was less than 0.05 and if *F* was greater than *F* crit. It was determined this was the case, so a Tukey-Kramer analysis was done on each paired interaction. This was done by calculating *d*, the absolute difference between averages calculated by ANOVA; denoting *n*, the number of replicates for each condition; calculating SE, the standard error using the equation: SE = square root(0.5 × MSω × (1/*n*_1_ + 1/*n*_2_)), where MSω = the mean square error within groups calculated by ANOVA, and *n*_1_ and *n*_2_ are the replicates for each condition; and calculating q, the *q* statistic as the absolute value of *d*/*SE* (Dr. Todd Grande, https://youtu.be/_l4O3xxh2ns).[1] Then, *q* was compared to a table of *q* values (https://www2.stat.duke.edu/courses/Spring98/sta110c/qtable.html and https://www.sjsu.edu/people/megumi.hosoda/courses/summer2013/s3/q-table.pdf)[2]. When *q* was greater than the *q* value from the table for α = 0.05, *df* (as determined by ANOVA for within groups), and the number of conditions (8 in this case), the null hypothesis was rejected and the data was considered statistically significant. An alternative calculation (https://youtu.be/P1j2CkOjWtM)[3] confirmed the results.

**References**

1. Grande T. Tukey-Kramer Post Hoc Test after One-Way ANOVA in Excel [cited 2021 10/12/2021]. Available from: <https://www.youtube.com/watch?v=_l4O3xxh2ns>.

2. Pearson E, Hartley H. Biometrika Tables for Statisticians. 3rd Ed. ed. New York: Cambridge University Press; 1966.

3. Design RB. Follow Up ANOVA - Tukey's HSD Post Hoc Test (12-5) [cited 2021 10/12/2021]. Available from: <https://www.youtube.com/watch?v=P1j2CkOjWtM>.
